# High prevalence of overweight, obesity, and hypertension with increased risk to cardiovascular disorders among adults in northwest Ethiopia: a cross sectional study

**DOI:** 10.1186/1471-2261-14-155

**Published:** 2014-11-05

**Authors:** Beyene Moges, Bemnet Amare, Bereket Fantahun, Afework Kassu

**Affiliations:** Department of Immunology and Molecular Biology, School of Biomedical and Laboratory Sciences, College of Medicine and Health Sciences, University of Gondar, Gondar, Ethiopia; Department of Biochemistry, School of Medicine, College of Medicine and Health Sciences, University of Gondar, Gondar, Ethiopia; Department of Pediatrics, Addis Ababa University, Addis Ababa, Ethiopia; Department of Medical Microbiology, School of Biomedical and Laboratory Sciences, College of Medicine and Health Sciences, University of Gondar, Gondar, Ethiopia

**Keywords:** Obesity, Overweight, Metabolic disorder, Hypertension, Anthropometry, Ethiopia

## Abstract

**Background:**

Overweight and obesity are components of a defined cluster of risk factors for non-communicable diseases, once problems for only the high-income countries, in recent days became rampant in developing countries. Despite the lack of extensive data on metabolic and cardio vascular disorders in Ethiopia, the prevalence of obesity among young adults (15–24 years), in a cross sectional study conducted in 1997, was 0.7% for men and 6% for women. The prevalence of hypertension (HTN) was found to be 7.1% of the population. The objective of this study was to see the prevalence and association of overweight, obesity and HTN and to check if there was any agreement among the various anthropometric measurements in detecting overweight and obesity.

**Methods:**

This cross-sectional study was conducted in Gondar city, Northwest Ethiopia. A total of 68 participants with age >18 year were randomly selected and included. Data were collected using questionnaires and through physical measurements of weight, height and blood pressure, using the WHO recommendations.

**Result:**

The prevalence of hypertension was 13.3% (9/68). The prevalence of overweight based on calculated body mass index (BMI) was 32.4% (22/68) while the prevalence of obesity was 16.2% (11/68). Body fat percentage (BFP) effectively classified all of the ‘overweight’ and ‘obese’ values according to the BMI as ‘overweight/obese’ (P = 0.016). Risk level classification with waist circumference enabled to correctly classify most (90.9%) and all of the ‘overweight’ and ‘obese’ BMI values as ‘increased risk/substantially increased risk’ (P < 0.001). Similarly, waist-to-height ratio (WHtR) was able to classify all ‘overweight’ and ‘obese’ BMI values as ‘increased risk/substantially increased risk’ (P < 0.001).

**Conclusion:**

In conclusion, the current study was able to detect a high prevalence of overweight, obesity, and HTN among adult population in Gondar town. There is a prevalent high level of general adiposity and central obesity. WHtR and BFP were the most efficient measurements to identify all ‘high risk’ groups of individuals as ‘high risk’ irrespective of their gender. Further study is recommended to elucidate the risk factors and complications of obesity and overweight in the study area and beyond.

## Background

Globally cardiovascular diseases account for approximately 17 million deaths a year, nearly one third of the total [1]. Of these, every year, complications of hypertension (HTN) account for 9.4 million deaths worldwide [2]. It is also believed that HTN is responsible for at least 45% and 51% of deaths due to heart disease and stroke, respectively [1]. In 2008, worldwide, approximately 40% of adults aged 25 and above had been diagnosed with HTN [3, 4].

According to the World Health Organization (WHO), the global prevalence of overweight and obesity in 2008 among adults was more than 1.4 billion and more than half a billion, respectively. It is believed that 65% of the world’s population lives in a country where overweight and obesity kills more people than underweight. Each year, it is estimated that at least 2.8 million people die as a result of being overweight or obese [5].

Overweight and obesity are components of a defined cluster of risk factors for non-communicable diseases, once problems for only the high-income countries, in recent days became rampant in developing countries. Major co-morbidities associated with these conditions include cardiovascular disease (CVD), cerebrovascular disease, type 2 diabetes mellitus (DM), atherogenic dyslipidaemia and certain types of cancer [6]. Globally, 44% of DM, 23% of ischaemic heart disease and 7–41% of certain cancers are attributable to overweight and obesity [5].

Despite the lack of extensive data on metabolic and CVD in Ethiopia, in 1997, a cross sectional study conducted in 1,436 (851 females and 585 males) young people and the prevalence of obesity was 0.7% for men and 6% for women [7]. The prevalence of HTN was found to be 7.1% of the population [7]. On the other hand, in 2008 a community-based study in Addis Ababa showed that 20% of men and 38% of women were overweight and 10.8% of these women were obese. Besides, 31.5% of men and 28.9% of women had HTN [8] indicating a silent but radical transition of metabolic and cardiovascular disorders in Ethiopia in the past few decades. The objective of this study was therefore to evaluate the prevalence and association of overweight, obesity and HTN and validate if there was any agreement among the various anthropometric measurements in detecting overweight and obesity.

Several anthropometric measures have been used to assess abnormal body fat distribution, including body mass index (BMI), waist circumference (WC) and waist-to-hip ratio (WHR) [9]. However, these measurements have been shown to correlate differently with CVD risk [10]. Even though BMI is used frequently as a measure of adiposity in epidemiologic studies, there is a claim that using BMI alone is not the most accurate measure of increased CVD risk rather WC and WHR [10]. On top of these, most of the existing recommendations are derived mainly from data obtained in Western populations underlining the necessity of country specific assessment of associations between measures of adiposity and CVD risk factors for prevention efforts.

## Methods

### Study design and subjects

This cross-sectional study was conducted in Gondar city, Northwest Ethiopia as a section of a dietary intake and nutritional status survey described elsewhere [11]. Gondar is a zonal capital city located 750kms north of Addis Ababa in Amhara Region. Based on the 2007 national census conducted by the Central Statistical Agency of Ethiopia, Gondar has a total population of 207,044, of whom 98,120 are men and 108,924 women. All participants with age >18 year and not acutely ill at the time of survey were randomly selected and included.

### Data collection

Data were collected using questionnaires and through physical measurements of weight, height and blood pressure (BP), using the WHO recommendations. Weight and height were measured with participants standing without shoes and wearing light clothing. Participants stood upright with the head in Frankfort plane for height measurement. Body weight (kg) was measured using an electronic scale to the nearest 10 g, and standing height was measured using a wall stadiometer to the nearest 0.1 cm. BMI was calculated as body weight (kg)/height (m^2^).

The subjects were then classified into four groups according to the WHO BMI cut-offs [9]:

“Underweight”: BMI <18.5 kg/m^2^

“Normal weight”: BMI =18.5 – 24.9 kg/m^2^

“Overweight”: BMI =25 – 29.9 kg/m^2^

“Obese”: BMI ≥30 kg/m^2^

Waist and hip circumferences were measured with a flexible steel metric tape at the nearest 0.5 cm. Waist circumference (WC) was measured midway between the lower rib margin and the iliac crest in the horizontal plane. While the subjects were standing, hip circumference (HC) was measured at the point yielding the maximum circumference over the buttocks using a tape measure to measure to the nearest 0.5 cm. Waist-to-hip ratio (WHR) was obtained by dividing the WC measurement with that of HC. Similarly, the waist-to-height ratio (WHtR) was obtained by dividing the WC value to the height of the individual.

Gender specific WC values denoting risk of metabolic complications [9]:

“Not increased”: <80 cm females, <94 cm males

“Increased”: 80–87.9 cm females, 94–101.9 cm males

“Substantially increased”: ≥88 cm females, ≥102 cm males

Gender specific WHR values denoting risk of metabolic complications [9]:

“Low risk” : <0.85 for females, <0.90 for males

“Increased risk” : > = 0.85 for females, > = 90 for males

WHtR values for both sexes denoting risk of metabolic complications [12]:

“Low adiposity” : <0.40

“Low risk”: 0.40-0.499

“Increased risk”: 0.50-0.59

“Substantially increased risk”: > = 0.60

Body fat composition (body fat percentage and visceral fat level) was assessed by means of Karada Scan™ Body Composition Monitor (Model HBF-358-BW, Omron healthcare Co., Ltd. Kyoto, Japan). The age and gender adjusted body fat percentage (BFP) cut off values are as summarized in the following table as calculated for African Americans by Gallagher et al. [13]:

The subjects were then classified into three groups based on the visceral fat level (VFL) using cut off values provided with Kaladar™ Scan”

“Low risk”: 1–9,

“High risk”: 10–14

“Very high risk”: 15–30

BP was measured, according to WHO guidelines, in a sitting position after the participant rested for at least 5 min. Three measurements were taken with intervals of 3 min between consecutive measurements. In addition, participants were asked whether they were taking any medications for the treatment of hypertension. Average systolic BP (SBP) and diastolic BP (DBP) were determined from the second and third measurements. Hypertension was defined as SBP > =140 mm Hg or DBP > =90 mm Hg or self reported use of antihypertensive medication, with adaptation of the recent WHO definitions [14].

### Ethical considerations

The study was conducted after ethical approval was obtained from Institutional Review Board of the University of Gondar and after informed consent was obtained from study participants. Participants with elevated blood pressure were advised and referred to the University of Gondar Hospital for proper management and follow up.

### Statistical analysis

Statistical Software for Social Sciences (SPSS) version 20 and Stata12 were used to analyze the data. Frequency distributions of sociodemographic characteristics and anthropometric measurements of the study population were determined by performing cross-tabulations of a variety of variables across gender and were expressed in percentage (%). Pearson’s chi square test was used to evaluate the differences in the distribution of categorical variables for study groups. The mean ± SD of anthropometric measurements were compared among men and women by one-way ANOVA. Post-hoc Tukey test was used to determine which pairs of means differ significantly. In all cases, P values less than 0.05 were considered statistically significant.

## Result

A total of 68 (Male = 29, Female = 39) adults were included in this study with mean (±SD, range) age of 38.76 (±10.72, 21–70) years (Table 1). Other sociodemographic characteristics and dietary intake pattern of the participants was published elsewhere [11]. The prevalence of HTN, based on raised SBP or DBP, was 13.3% (9/68). The prevalence among men and women was 17.2% (5/29) and 10.3% (4/39), respectively. On the other hand, the prevalence of pre-HTN (SBP > =130 mm Hg or DBP > =80 mm Hg) 38.2% (26/68).

**Table 1:** **Age and gender adjusted BFP cut off values as estimated for African Americans**

Age (years)	BMI (kg/m ^2^)	Body fat (%)	
		Female	Male
**20-39**	<18.5	<20	<8
	18.5-24.9	20-31.9	8-19.9
	25-29.9	32-37.9	20-25.9
	> = 30	> = 38	> = 26
**40-59**	<18.5	<21	<9
	18.5-24.9	21-33.9	9-21.9
	25-29.9	34-38.9	22-26.9
	> = 30	> = 39	> = 27
**60-79**	<18.5	<23	<11
	18.5-24.9	23-34.9	11-22.9
	25-29.9	35-40.9	23-28.9
	> = 30	> = 41	> = 29

Table 2 summarizes the various anthropometric and blood pressure measurements of the participants based on their gender. While the prevalence of overweight in the current study based on measurements of the BMI was 32.4% (22/68), the prevalence of obesity was 16.2% (11/68).

**Table 2:** **Distribution of age groups and anthropometric measurements tabulated against sex of participants, N = 68 (M = 29, F = 39), Gondar, Ethiopia**

		Sex			P value
		Male N (%)	Female N (%)	Total (%)	
Age (years)	20-39	15 (51.7)	21 (53.8)	36 (52.9)	0.69
	40-59	12 (41.4)	17 (43.6)	29 (42.6)	
	60-79	2 (6.9)	1 (2.6)	3 (4.4)	
BMI	Underweight	1 (3.4)	1 (2.6)	2 (2.9)	0.733
	Normal	15 (51.7)	18 (46.2)	33 (48.5)	
	Overweight	10 (34.5)	12 (30.8)	22 (32.4)	
	Obese	3 (10.3)	8 (20.5)	11 (16.2)	
WC	Low risk	14 (48.3)	4 (10.3)	18 (26.5)	0.000
	Increased risk	9 (31)	6 (15.4)	15 (22)	
	Substantially increased risk	6 (20.7)	29 (74.4)	35 (51.5)	
WHR	Low risk	9 (31)	9 (23.1)	18 (26.5)	0.462
	Substantially increased risk	20 (69)	30 (76.9)	50 (73.5)	
WHtR	Low risk	5 (17.2)	3 (7.7)	8 (11.8)	0.014
	Increased risk	19 (65.6)	16 (41)	35 (51.5)	
	Substantially increased risk	5 (17.2)	20 (51.3)	25 (36.7)	
BFP*	Normal	5 (17.2)	5 (12.8)	10 (14.7)	0.011
	Over weight	6 (20.7)	22 (56.4)	28 (41.2)	
	Obese	18 (62.1)	12 (30.8)	30 (44.1)	
VFL	Low risk	12 (41.4)	28 (71.8)	40 (58.8)	0.018
	High risk	12 (41.4)	5 (12.8)	17 (25)	
	Very high risk	5 (17.2)	6 (15.4)	11 (16.2)	
SBP	Normal	17 (58.8)	26 (66.7)	43 (63.2)	0.672
	Pre-HTN	8 (27.6)	10 (25.6)	18 (26.5)	
	HTN	4 (13.8)	3 (7.7)	7 (10.3)	
DBP	Normal	13 (44.8)	31 (79.5)	44 (64.7)	0.013
	Pre- HTN	12 (41.4)	6 (15.4)	18 (26.5)	
	HTN	4 (13.8)	2 (5.1)	6 (8.8)	

*The sex adjusted BFP cut off values were considered for each age group.

DBP = diastolic blood pressure, SBP = systolic blood pressure, HTN = hypertension, BFP = Body fat percentage, BMI = body mass index, WC = waist circumference, WHR = waist-to-hip ratio, WHtR = Waist-to-height ratio, VFL = visceral fat level, N = number, M = male, F = female.

Based on the measurements from the WC of individual participants the risk of developing CVDs was rated to be increased in 22% (15/68) and substantially increased in 51.5% (35/68). Similarly, based on WHR 73.5% (50/68) of the individuals were found to have increased risks of developing CVDs. Risk classification with WHtR showed that 51.5% (35/68) and 36.7% (25/68) of the participants had high risk and substantially increased risk, respectively. Risk level according to VFL was found to be high in 25% (17/68) and very high in 16.2% (11/68) (Table 2).

Table 3 describes the Mean ± SD (95% CI) of the various anthropometric measurements of participants by sex. The mean BMI (Kg/m^2^) of the participants was generally higher in the study participants, 25.26 ± 4.41 (95% CI, 24.2-26.3). The mean WC and WHR of the participants were 94.26 ± 10.72 (95% CI, 91.67-96.86) and 0.91 ± 0.07 (95% CI, 0.89-0.93), respectively. On the other hand, the mean WHtR was general high (0.579 ± 0.073) with a significant intersex variation (P = 0.008) (Table 3). There was a significant mean BFP variation among men and women, as expected. It was found out to be 26.36 ± 6.00 (95% CI, 24.08-28.64) and 36.51 ± 4.50 (95% CI, 35.05-37.97) for men and women, respectively (P < 0.001) (Table 3).

**Table 3:** **Mean ± SD (95%CI) of various anthropometric measurements of participants by sex, N = 68 (M = 29, F = 39), Gondar, Ethiopia**

Parameter	Male	Female	Total	P value
	Mean ± SD (95% CI)	Mean ± SD (95% CI)	Mean ± SD (95% CI)	
**Weight (kg)**	70.49 ± 10.88 (66.35-74.63)	64.62 ± 12.14 (60.68-68.55)	67.12 ± 11.9 (64.24-70.0)	0.043
**Height (cm)**	169.52 ± 7.27 (166.75-172.28)	158.28 ± 4.9 (156.69-159.87)	163.07 ± 8.19 (161.09-165.06)	0.000
**BMI (kg/m** ^**2**^ **)**	24.59 ± 3.99 (23.07-26.11)	25.77 ± 4.68 (24.25-27.29)	25.27 ± 4.4 (24.2-26.3)	0.277
**WC (cm)**	93.55 ± 9.21 (90.05-97.05)	94.79 ± 11.81 (90.97-98.62)	94.26 ± 10.72 (91.67-96.86)	0.640
**WHR**	0.92 ± 0.05 (0.90-0.94)	0.90 ± 0.08 (0.87-0.93)	0.91 ± 0.07 (0.89-0.93)	0.253
**WHtR**	0.553 ± 0.059 (0.53-0.575)	0.599 ± 0.077 (0.574-0.624)	0.579 ± 0.073 (0.562-0.597)	0.008
**BFP**	26.36 ± 6.00 (24.08-28.64)	36.51 ± 4.50 (35.05-37.97)	32.18 ± 7.2 2 (30.43-33.93)	0.000
**VFL**	10.41 ± 5.24 (8.42-12.41)	8.26 ± 5.41 (6.50-10.01)	9.18 ± 5.41 (7.87-10.49)	0.104
**SBP (mm Hg)**	122.1 ± 18.4 (115.1-129.1)	115 ± 16.1 (110–120)	118 ± 17.3 (114–122.2)	0.092
**DBP (mm Hg)**	80.8 ± 11.2 (76.5-85)	74.1 ± 10.4 (70.8-77.5)	77 ± 11.2 (74.27-79.7)	0.014

DBP = diastolic blood pressure, SBP = systolic blood pressure, HTN = hypertension, BFP = Body fat percentage, BMI = body mass index, WC = waist circumference, WHR = waist-to-hip ratio, WHtR = Waist-to-height ratio, VFL = visceral fat level, CVDs = cardiovascular disorders, N = number, M = male, F = female, SD = standard deviation, CI = confidence interval.

The mean SBP and DBP of the participants were in the normal ranges, 118 ± 17.3 (95% CI, 114–122.2) and 77 ± 11.2 (95% CI, 74.27-79.7), respectively (Table 3). Table 4 also describes the means of the BFP of participants based on specific age and sex groups in comparison to the normal range. In all the sex adjusted age groups, the mean BFP was found to be higher than the respective normal ranges/cut off values.

**Table 4:** **Mean ± SD (95%CI) of the body fat percentage of participants in comparison to the normal range based on sex and age groups, Gondar, Ethiopia**

Age (years)			Body fat percentage	
	Current study		Normal range	
	Male*	Female**	Male	Female
**20-39**	24.84 ± 7.38 (20.75-28.93)	34.57 ± 4.45 (32.55-36.58)	8-20	20-32
**40-59**	27.61 ± 3.74 (25.23-29.99)	38.54 ± 3.41 (36.78-40.29)	9-22	21-34
**60-79**	30.25 ± 2.90 (4.20-56.30)	42.70***	11-23	23-35

*P value = 0.325.

**P value = 0.007.

***Unable to compete SD and CI because there was only one participant in this age group.

SD = standard deviation, CI = confidence interval.

Table 5 presents the classification of nutritional status and risk level to CVDs using different anthropometric measurements and compares them against BMI. Accordingly, BFP effectively classified 100% (22/22) of the ‘overweight’ and 100% (11/11) of the ‘obese’ values according to the BMI as ‘overweight/obese’ and ‘overweight/obese’, respectively (P = 0.016). On the other hand, VFL was able to correctly classify 63.6% (14/22) of the ‘overweight’ and 90.9% (10/11) ‘obese’ values as ‘increased risk/substantially increased risk’ (P < 0.001) (Table 5).

**Table 5:** **Classification of nutritional status using and comparison of risk level to CVDs using BFP, VFL, WC, and WHR against BMI, N = 68 (M = 29, F = 39), Gondar, Ethiopia**

	BMI				
	Underweight N (%)	Normal N (%)	Over weight N (%)	Obese N (%)	Total N (%)
BFP* Normal	1 (50)	9 (27.3)	0	0	10 (14.7)
Overweight	1 (50)	14 (42.4)	10 (45.5)	3 (27.3)	28 (41.2)
Obese	0	10 (30.3)	12 (54.5)	8 (72.7)	30 (44.1)
Total N (%)	2 (2.9)	33 (48.5)	22 (32.4)	11 (16.2)	68 (100)
VFL** Low risk	2 (100)	29 (87.9)	8 (36.4)	1 (9.1)	40 (58.8)
Increased risk	0	4 (12.1)	11 (50)	2 (18.2)	17 (25)
Very increased risk	0	0	3 (13.6)	8 (72.7)	11 (16.2)
Total	2 (2.9)	33 (48.5)	22 (32.4)	11 (16.2)	68 (100)
WC (cm)*** Low risk	2 (100)	14 (42.4)	2 (9.1)	0	18 (26.5)
Increased risk	0	8 (24.3)	7 (31.8)	0	15 (22)
Substantially increased risk	0	11 (33.3)	13 (59.1)	11 (100)	35 (51.5)
Total N (%)	2 (2.9)	33 (48.5)	22 (32.4)	11 (16.2)	68 (100)
WHR**** Low risk	2 (100)	8 (24.4)	7 (31.8)	1 (9.1)	18 (26.5)
Increased risk	0	25 (75.6)	15 (68.2)	10 (90.9)	50 (73.5)
Total	2 (2.9)	33 (48.5)	22 (32.4)	11 (16.2)	68 (100)
WHtR***** Low risk	2 (100)	6 (18.2)	0	0	8 (11.8)
Increased risk	0	22 (66.7)	13 (59.1)	0	35 (51.5)
Substantially increased risk	0	5 (15.1)	9 (40.9)	11 (100)	25 (36.8)
Total N (%)	2 (2.9)	33 (48.5)	22 (32.4)	11 (16.2)	68 (100)

*Pearson’s Chi Square = 15.65, P = 0.016 , **Pearson’s chi square = 47.71, P = 0.000, ***Pearson’s, chi square = 24.99, P = 0.000, ****Pearson’s chi square = 7.67, P = 0.053, *****Pearson’s chi square = 43.69, P = 0.000.

BFP = Body fat percentage, BMI = body mass index, WC = waist circumference, WHR = waist-to-hip ratio, WHtR = Waist-to-height ratio, VFL = visceral fat level, CVDs = cardiovascular disorders, N = number, M = male, F = female.

Risk level classification for CVD with WC enabled to correctly classify 90.9% (20/22) and 100% (11/11) of the ‘overweight’ and ‘obese’ BMI values as ‘increased risk/substantially increased risk’ and ‘substantially increased’, respectively (P < 0.001) (Table 5).

Risk level classification with WHR was effective in classifying 68.2% (15/22) and 90.9% of ‘overweight’ and ‘obese’ BMI cases as ‘increased risk’ and ‘increased risk’, respectively (P = 0.053). On the other end, WHtR was able to classify 100% (22/22) ‘overweight’ BMI values as ‘increased risk/substantially increased risk’. It also classified 100% (11/11) ‘obese’ BMI values as ‘substantially increased risk’ (P < 0.001) (Table 5).

Table 6 presents risk level to CVDs and compares WC, BFP, VFL, and WHtR with WHR values of participants. Waist circumference was able to effectively categorize 88% (44/50) of the ‘increased risk’ WHR values as ‘increased risk/substantially increased risk’ (P < 0.001). Likewise, BFP was able to correctly classify 90% (45/50) of the ‘increased risk/substantially increased’ WHR values as ‘overweight/obese’ (P = 0.051). On the other hand, WHtR was able to identify 100% (50/50) ‘increased risk’ WHR values as ‘increased risk/substantially increased risk’ (P < 0.001) (Table 6).

**Table 6:** **Comparison of risk level to CVDs using WC, BFP and VFL against WHR of participants, N = 68 (M = 29, F = 39), Gondar, Ethiopia**

	WHR		
	Low risk N (%)	Increased risk N (%)	Total N (%)
WC (cm)* Low risk	12 (66.6)	6 (12)	18 (26.5)
Increased risk	3 (16.7)	12 (24)	15 (22)
Substantially increased risk	3 (16.7)	32 (64)	35 (51.5)
Total N (%)	18 (26.5)	50 (73.5)	68 (100)
WHtR** Low risk	8 (44.4)	0	8 (11.8)
Increased risk	8 (44.4)	27 (54)	35 (51.5)
Substantially increased risk	2 (11.2)	23 (46)	25 (36.8)
Total N (%)	18 (26.5)	50 (73.5)	68 (100)
BFP*** Normal	5 (27.8)	5 (10)	10 (14.7)
Overweight	9 (50)	19 (38)	28 (41.2)
Obese	4 (22.2)	26 (52)	30 (44.1)
Total	18 (26.5)	50 (73.5)	68 (100)
VFL**** Low risk	13 (72.2)	27 (54)	40 (58.8)
High risk	4 (22.2)	13 (26)	17 (25)
Very high risk	1 (5.6)	10 (20)	11 (16.2)
Total N (%)	18 (26.5)	50 (73.5)	68 (100)

*Pearson’s chi square = 21.03, P = 0.000.

**Pearson’s chi square = 26.84, P = 0.000.

***Pearson’s chi square = 5.97, P = 0.051.

****Pearson’s chi square = 21.03, P = 0.282.

BFP = Body fat percentage, WC = waist circumference, WHR = Waist-to-hip ratio, WHtR = Waist-to-height ratio, VFL = Visceral fat level, CVDs = Cardiovascular disorders, N = number, M = male, F = female.

Table 7 presents risk level to CVDs and compares BFP and VFL with WC values of participants. BFP was able to effectively categorize 93.3% (14/15) and 94.3% (33/35) of ‘increased risk’ and ‘substantially increased risk’ WC values as ‘overweight/obese’ and ‘overweight/obese’, respectively (P < 0.001). VFL, on the contrary, detected only 46.7% (7/15) and 48.6% (17/35) of the ‘increased risk’ and ‘substantially increased risk’ WC values as ‘high risk/very high risk’ (P = 0.035).

**Table 7:** **Comparison of risk level to CVDs using BFP and VFL against WC, N = 68 (M = 29, F = 39), Gondar, Ethiopia**

	WC (cm)			
	Low risk N (%)	Increased risk N (%)	Substantially increased risk	Total N (%)
BFP* Normal	7 (38.9)	1 (6.7)	2 (5.7)	10 (14.7)
Overweight	5 (27.8)	8 (53.3)	15 (42.9)	28 (41.2)
Obese	6 (33.3)	6 (40)	18 (51.4)	30 (44.1)
Total N (%)	18 (26.5)	15 (22.1)	35 (51.5)	68 (100)
VFL** Low risk	14 (77.8)	8 (53.3)	18 (51.4)	40 (58.8)
High risk	4 (22.2)	6 (40)	7 (20)	17 (25)
Very high risk	0	1 (6.7)	10 (28.6)	11 (16.2)
Total N (%)	18 (26.5)	15 (22.1)	35 (51.5)	68 (100)
WHtR^***^ Low risk	8 (44.4)	0	0	8 (11.8)
Increased risk	10 (55.6)	15 (100)	10 (28.6)	35 (51.5)
Substantially increased risk	0	0	25 (71.4)	25 (36.7)
Total N (%)	18 (26.5)	15 (22.1)	35 (51.5)	68 (100)

*Pearson’s chi square = 12.05, P = 0.017.

**Pearson’s chi square = 10.33, P = 0.035.

***Pearson’s chi square = 56.28, P = 0.000.

BFP = Body fat percentage, WC = Waist circumference, WHtR = Waist-to-height ratio, VFL = Visceral fat level, CVDs = Cardiovascular disorders, N = number, M = male, F = female.

## Discussion

This study was conducted to evaluate the situation of HTN and metabolic abnormalities (based on anthropometric measurements only) as a risk factor for CVDs among 68 adults in Gondar Town, Ethiopia. Accordingly, the prevalence of HTN was 13.3%; 17.2% among men and 10.3% among women based on raised SBP (> = 140 mm Hg) and DBP (> = 9 0 mm Hg). A study conducted among adults in Addis Ababa reported a prevalence of 31.5% for men and 28.9% for women [8] which is much higher than found in this study. Similarly, the WHO estimated the prevalence of HTN in Ethiopia for the year 2008 to be 35.2%, way too much higher than in the current study. The estimates for sex adjusted prevalence were also much higher, 37.3% and 33.2% among males and females, respectively [15]. Nevertheless, the prevalence of HTN in the current study is well above that of a study conducted in the mid 1990s which reported a prevalence of 7.1% even though the study was conducted among young adults only [7]. The current finding underscores the need of further survey incorporating large segment of the population. The increasing prevalence of HTN, as frequently reported, could be due to unhealthy diet, harmful use of alcohol, lack of physical activity, excess weight and exposure to persistent stress [16–19].

A rather intriguing finding in the current study was, according to BMI measurements, a high prevalence of overweight (32.4%) and obesity (16.2%). The sex adjusted prevalence of overweight was also very high (34.5% for men and 30.8% for women) with small variation (P > 0.05). On the other hand, the prevalence of obesity among men and women in the current study were 10.3% and 20.5%. There were more obese women but the variation was not statistically significant (P > 0.05). According to the WHO, the estimated prevalence of overweight was 7.4% in 2008 much lower than the figures in this study. Similarly, the estimates for men and women were also much lower with 6.2% and 8.6%, respectively. The estimated prevalence of obesity was also by far very low according to the WHO, 1.1%; only 0.7% among men and 1.5% among women [15]. On the other hand, in comparison to the report in Addis Ababa (20.2% for men and 37.7% for women), overweight was more prevalent in Gondar among men and less prevalent among women [8]. The prevalence of overweight in the current study was several times higher than the one reported from three demographic surveillance sites in Ethiopia (2.5 and 2.2% among men and women) [20].

These figures will be more intriguing when nutritional status is defined based on the total body fat percentage (BFP) which reported a 41.2% of overweight and 44.1% of obesity in the study population. The BFP classified more men as obese (62.1%) than women (30.8%) whereas it classified more women as overweigh (56.4%) than men (20.7%). There was a significant nutritional status variation across gender (P = 0.011) (Table 3). Even though the current study was conducted in a smaller section of randomly selected adults, the figures are not quite ignorable. Hence, demand further study and intervention.

There are a number of grave and chronic consequences (CVDs including HTN, DM and cancer) of being overweight or obese. Sixty-three per cent (63%) of global deaths in 2008 (i.e. 36 million of the 57 million global deaths) resulted from non communicable diseases, principally CVDs, DM, and cancers [21]. To make matters worse, nearly 80% (28 million) of these deaths were believed to have occurred in low- and middle-income countries. Therefore, underlying causes/factors and potential associated risks of the prevalent obesity and overweight in the current study require fair attention to plan a proper intervention.

Even though BMI is commonly used as a measure of overall adiposity and classify risk level to various chronic illnesses [22–25], growing evidence suggests that a central (abdominal) fat distribution pattern, as reflected by a higher WC or WHR might be a better measure of risk [26–34]. In this study, based on measurements of WC, the risk of developing CVDs was rated to be increased or substantially increased (men >94 cm and >102 cm; women >80 cm and >88 cm) in more than half of the men (51.7%) and majority of the women (with women more likely to have central obesity than men, P < 0.001). On the other hand, based on measurements of WHR, 69% of men and 76.9% of women were found to have increased risks (men > = 0.90, women > = 0.85) of developing CVDs without significant difference across gender (P = 0.462). This quite high figure demands a large scale study to define the cut off values.

There is growing evidence to use the WHTR as a more sensitive measurement than BMI as an early warning of health risks [35, 36]. It is also reported WHTR could be more closely associated with central obesity than BMI [37] and even better than WC as it encompasses the adjustment to different statures [38, 39]. The risk level in the current study was rated to be either increased (WHtR > = 0.50) or substantially increased (WHtR > = 0.60) in 88.2% of the participants. The risk level across gender was also increased/substantially increased with WHtR of 82.8% and 92.3% among men and women, respectively, with women to have more central obesity than men (0.014). On the contrary, general risk level according to VFL was found to be either increased or substantially increased in 41.2%; 58.6% men and 28.2% women (p = 0.018).

In the current study, the sex adjusted mean SBP (mm Hg) was 122.1 (115.1-129.1) and 115 (110–120) among men and women, respectively which is much lower in comparison to the one reported in Addis Ababa which showed a mean SBP of 129.4 mmHg (128.4-130.5) among males and 126.2 mmHg (125.1-127.2) among females [8]. On the other hand, the mean DBP (mmHg) in the current study among men 80.8 (76.5-85) was in agreement the one reported in Addis Ababa 81.2 mmHg (80.6-81.9) [8]. However, the mean DBP (mm Hg) among women 74.1 (70.8-77.5) in this study was lower than that of the report from Addis Ababa 80.0 mmHg (79.5-80.6) [8]. On the contrary, the mean SBP and DBP in the current study were higher than reported elsewhere in Ethiopia [20].

In this study, it was observed that the mean BMI (Kg/m^2^) (95% CI) of the participants was generally higher, 25.26 (24.2-26.3). There was not significant variation of mean BMI (kg/m^2^) among men and women (0.227), 24.59 (23.07-26.11) and 25.77 (24.25-27.29), respectively. However, the mean remained high. On the contrary, there was a significant variation in the mean weights and heights of women and men. The mean (95% CI) of weight (kg) was calculated to be 70.49 (66.35-74.63) and 64.62 (60.68-68.55) for men and women, respectively (P = 0.043). Similarly, the mean (95% CI) height (cm) was computed to be 169.52 (166.75-172.28) and 158.28 (156.69-159.87) for men and women, respectively (P < 0.001). The mean BMI (kg/m^2^) and weight (kg) in the current study were much higher than the respective figures reported elsewhere in Ethiopia while the mean height (cm) was in harmony [20].

Another interesting finding from this study was a higher mean (95% CI) WC of the participants 94.26 (91.67-96.86). Even if the mean WC of the participants didn’t significantly vary among sex groups, [93.55 (90.05-97.05) and 94.79 (90.97-98.62) among men and women, respectively] the mean value for women was much higher than the respective cut off. On the other hand, the mean WHtR was generally high (0.579) with a significant intersex variation (P = 0.008). Likewise, the mean age adjusted BFP was higher than the expected cut off values for both men. Besides, there was a significant mean BFP variation across the three age groups among women (P = 0.007).

In this study, it was attempted to compare the various anthropometric measurements against BMI. Even though there is not one gold standard anthropometric measurement to classify risk level, BMI, WC and WHR were used as ‘standard’. Accordingly, BFP was able to effectively classify all of the ‘overweight’ and the ‘obese’ values based on the BMI as ‘overweight/obese’. Besides, BFP was able to detect 25 additional ‘overweight or obese’ cases missed by the BMI (P = 0.016). Likewise, risk level classification with WC was able to correctly classify most (20/22) of the ‘overweight’ and all of the ‘obese’ BMI values as ‘increased risk and/or substantially increased risk. It was also able to detect 19 more ‘increased risk and/or substantially increased’ cases (P < 0.001) than the BMI. Similarly, WHtR was able to classify all the ‘overweight’ and ‘obese’ BMI values as ‘increased risk and/or substantially increased risk’. In addition to this, it was able to detect 27 more ‘increased risk and/or substantially increased risk’ cases missed by the BMI (P < 0.001).

On the contrary, VFL was able to correctly classify only 63.6% (14/22) of the ‘overweight’ and 90.9% (10/11) ‘obese’ values as ‘increased risk/substantially increased risk’. Apart from this, VFL misclassified 9 ‘overweight/obese’ cases detected by the BMI (P < 0.001). Similarly, risk level classification with WHR was effective in classifying only 68.2% (15/22) and 90.9% (10/11) of ‘overweight’ and ‘obese’ BMI cases as ‘increased risk’. It also missed 8 ‘overweight/obese’ cases (P = 0.053) detected by the BMI. It is evident that in all of the anthropometric measurements compared with BMI, they were able to detect nearly all obese cases. Nevertheless, further study is required to substantiate this assumption. On the other hand, WHtR, BFP, WC, and VFL were able to effectively categorize 50, 45, 44, and 23 of the 50 ‘increased risk’ WHR values as ‘increased risk/substantially increased risk’, respectively. Besides, WHtR, BFP, WC, and VFL were able to detect 8, 13, 3, and 5 additional ‘increased risk/substantially increased risk’ cases, respectively.

When WHtR, BFP and VFL were compared with WC values, WHtR was able to correctly classify all ‘increased risk/substantially increased’ WC values. It also detected 10 additional ‘increased risk’ cases (P < 0.001). On the other hand, BFP was able to effectively categorize 14/15 and 33/35 of ‘increased risk’ and ‘substantially increased risk’ WC values as ‘overweight/obese’ (P < 0.001). VFL, on the contrary, detected only 7/15) and 17/35 of the ‘increased risk’ and ‘substantially increased risk’ WC values as ‘high risk/very high risk’ (P = 0.035). So far, WHtR seems to be the most efficient anthropometric measurement to strongly agree with the other anthropometric measurements though large scale studies are required to substantiate this finding.

The current study couldn’t outline whether certain anthropometric values were associated with HTN, DM, cancer, or other chronic diseases and biochemical parameters were not collected as well. This obviates the need of a longitudinal study in the country.

The small sample size was a major limitation of the study; results must be interpreted with caution. The study was carried out in only one city of Northwest Ethiopia and thus the finding cannot be generalized to HTN in Ethiopia. Its cross-sectional design was also limited in evaluating cause-and-effect associations.

## Conclusion

In conclusion, the current study was able to detect a high prevalence of overweight, obesity, and HTN among adult population in Gondar town. The mean BMI of the participants was in the range of overweight values. There is a prevalent high level of general adiposity evidenced by the high BFP in all age groups and sexes. The central obesity as measured by WC, WHR, WHtR and VFL was also very high. Even though there is a need of large scale study in the country to set national cut off values to efficiently level risk groups, WHtR and BFP were the most efficient measurements to identify nearly all ‘high risk’ groups of individuals as ‘high risk’ irrespective of their gender. The VFL, in contrary, was unable to identify quite a significant number of ‘high risk’ groups. In general, both general adiposity, captured by BMI or BFP, and abdominal adiposity, captured by WC, WHR, WHtR or VFL could independently be risk factors for certain diseases. Therefore, it is important to elucidate the mechanisms and independent roles of body fat distribution on the etiology of chronic diseases.

Despite the fact that the study wasn’t conducted on a large sample size population and evenly distributed age and sex groups, the findings of the study can alarm that a salient but an unnoticed epidemics of non-communicable diseases in northwest Ethiopia and beyond. Even if the most important risk factors could be similar with other populations in the world, it is mandatory to specifically identify the most important risk factors in order to plan a public health control program.

## Abbreviations

BFP: Body fat percentage
BMI: Body mass index
BP: Blood pressure
CI: Confidence interval
CVDs: Cardiovascular disorders
DBP: Diastolic pressure
DM: Diabetes mellitus
HC: Hip circumference
HTN: Hypertension
SBP: Systolic blood pressure
SPSS: Statistical package for social sciences
SD: Standard deviation
WHO: World health organization
WHR: Waist-to-hip ratio
WC: Waist circumference
WHtR: Waist-to-height ratio
VFL: Visceral fat level.
